# Rate of Memory Change Before and After Cancer Diagnosis

**DOI:** 10.1001/jamanetworkopen.2019.6160

**Published:** 2019-06-21

**Authors:** Monica Ospina-Romero, Ekland Abdiwahab, Lindsay Kobayashi, Teresa Filshtein, Willa D. Brenowitz, Elizabeth R. Mayeda, M. Maria Glymour

**Affiliations:** 1 Department of Epidemiology and Biostatistics, University of California, San Francisco; 2 Lombardi Comprehensive Cancer Center, Georgetown University, Washington, DC; 3 Department of Epidemiology, Jonathan and Karin Fielding School of Public Health, University of California, Los Angeles

## Abstract

**Importance:**

Patients with a history of cancer, even nonfatal cancers, have lower subsequent Alzheimer disease incidence. An inverse biological link between carcinogenesis and neurodegeneration has been hypothesized, although survival and detection biases are possible explanations.

**Objective:**

To compare long-term memory trajectories before and after incident cancer with memory trajectories of similarly aged individuals not diagnosed with cancer.

**Design, Setting, and Participants:**

This population-based cohort study included 14 583 US adults born before 1949 with no cancer history from the Health and Retirement Study. Biennial assessments were performed for up to 16 years from 1998 to 2014. Data analysis was performed from January 8 to October 5, 2018.

**Exposures:**

Self-reported physician diagnosis of any cancer (excluding nonmelanoma skin cancer) during follow-up.

**Main Outcomes and Measures:**

A composite memory score standardized to a mean (SD) of 0 (1) at baseline was based on immediate and delayed word-list recall and proxy assessments. The rate of memory change among people diagnosed with cancer during follow-up before and after diagnosis was compared with rate of memory change in individuals who remained cancer free during follow-up using linear mixed-effect models with random intercepts and slopes.

**Results:**

A total of 14 583 participants were included in the sample (mean [SD] age, 66.4 [10.4] years; 8453 [58.0%] female). The mean (SD) follow-up was 11.5 (5.1) years; 2250 had a cancer diagnosis during follow-up, and 12 333 had no cancer diagnosis during follow-up. The rate of memory decline in the decade before a cancer diagnosis was 10.5% (95% CI, 6.2%-14.9%), which was slower than memory decline in similarly aged cancer-free individuals. For individuals diagnosed at 75 years of age, mean memory function immediately before diagnosis was 0.096 SD units (95% CI, 0.060-0.133 SD units) higher compared with that among similarly aged cancer-free individuals. A new cancer diagnosis was associated with a short-term decline in memory of −0.058 (95% CI, −0.084 to −0.032) SD units compared with memory before diagnosis. After diagnosis, the rate of memory decline was 3.9% (95% CI, 0.9%-6.9%) slower in individuals with cancer than in those without a cancer diagnosis.

**Conclusions and Relevance:**

In this study, older individuals who developed cancer had better memory and slower memory decline than did similarly aged individuals who remained cancer free. These findings support the possibility of a common pathologic process working in opposite directions in cancer and Alzheimer disease.

## Key Points

**Question:**

Are factors associated with carcinogenesis associated with a slower decline in memory function before and after cancer diagnosis in middle-aged and older US adults?

**Findings:**

In this population-based cohort study of 14 583 individuals, those with an incident cancer had modestly higher memory function and slower memory decline both before and after their diagnosis than similarly aged individuals who remained cancer free for a mean 11.5-year follow-up.

**Meaning:**

Factors associated with carcinogenesis may also be associated with cognitive impairment and Alzheimer disease.

## Teaser

This cohort study compares long-term memory changes before and after incident cancer diagnoses in US adults compared with similarly aged adults without a diagnosis of cancer.

## Introduction

Many observational studies^1,2,3,4,5^ have reported lower incidence of Alzheimer disease (AD) among individuals with a history of cancer compared with cancer-free population controls. This association has been observed for nearly all cancer types examined to date, including smoking- and non–smoking-related cancers as well as nonfatal cancers, such as nonmelanoma skin cancer.^4,6,7^ Biological explanations for this association typically invoke the concept that carcinogenesis and neurodegeneration may be at opposite ends of a common pathologic process.^8,9^ Research has shown lower density of neurofibrillary tangles at autopsy in individuals with a history of cancer than in those who never received a cancer diagnosis.^10^ Mechanisms of cancer that are targets of diverse drugs have multiple points of overlap (in an opposing direction) with hypothesized pathologic pathways of AD (eg, regulation of the cell cycle and apoptosis, innate immune function, cellular metabolism, and DNA repair mechanisms).^11,12^ The enzyme PIN1 illustrates potential mechanisms; PIN1 is upregulated in numerous human cancers but downregulated in AD.^12^ PIN1 catalyzes isomerization of pSer/Thr-Pro motifs to influence multiple cellular processes, including activation of telomerase and genomic repair by preserving p53 functioning, ultimately regulating cell cycle processes. PIN1 is also believed to suppress tau and amyloid β deposition in AD.^13^

An alternative explanation for the inverse association between cancer and AD is that individuals who survive long enough to develop cancer represent a select group of healthy individuals and that the same factors that promote cancer survival may also protect against AD.^8^ Consequently, selective survival of healthy individuals could generate a spurious association between history of cancer and AD incidence.

The inverse association between cancer and AD is especially puzzling because neuropsychological studies^14,15^ of patients with malignant conditions, such as breast and prostate cancers, suggest that receipt of chemotherapy is associated with a well-characterized, short-term cognitive decline. Multidomain cognitive impairment is now considered to be a potential adverse effect of cytotoxic cancer therapies,^16^ and in 2011, the International Cognition and Cancer Task Force^17^ recommended studies with repeated cognitive assessments over time to understand the time course and mechanisms of cancer-associated cognitive deficits.

Currently, little evidence is available on long-term cognitive trajectories in individuals with a new cancer diagnosis vs those with no cancer history, although such evidence may help to clarify these prior findings. The continuum of mild cognitive impairment and AD is preceded by several years of accelerated cognitive loss.^18,19^ Repeated measurements of cognitive functioning over time can detect early stages of AD and are less susceptible to detection bias and survival bias compared with studies that examine clinical diagnosis of dementia as the outcome.

Population-based evidence from individuals with incident cancer diagnosis and individuals who remain free of cancer may reveal whether differences in cognitive function between these 2 groups are apparent before cancer diagnosis. For example, comparing cognitive trajectories before an incident cancer diagnosis in adults with and without cancer would aid in understanding of the role of cancer in altering cognitive change over time during aging. Evaluating cognitive trajectories before cancer diagnosis may also help to avoid any potential bias attributable to differential survival after cancer diagnosis. If evidence is found for favorable cognitive performance in patients with cancer compared with cancer-free population controls in the period preceding the cancer diagnosis, independently of any potential confounding factor, this evidence may support the hypothesis of a common pathologic pathway between carcinogenesis and neurodegeneration. Repeated assessments of cognitive function during the years before and after cancer diagnosis compared with cancer-free population-based controls may allow detection of any change in cognitive function immediately after cancer diagnosis and the potential changes in the slopes of cognitive change after a new cancer diagnosis is established. We therefore compared long-term memory trajectories in individuals before and after a new cancer diagnosis with aging-related memory trajectories in a US population–based cohort of individuals with no cancer diagnosis.

## Methods

### Study Design and Population

This population-based cohort study included US adults born before 1949 with no cancer history from the Health and Retirement Study (HRS). The HRS cohort was drawn from a target population of middle-aged or older (≥50 years of age) noninstitutionalized adults in the contiguous United States. Data sets are listed in the eAppendix 1 in the Supplement. The HRS was sponsored by the National Institute on Aging, National Institutes of Health, and is conducted by the University of Michigan, Ann Arbor. All participants in the study gave verbal informed consent for their participation. All data were deidentified. The HRS was approved by the institutional review board at the University of Michigan. The current study, as a secondary analysis of deidentified and publicly available HRS data, was certified as exempt from review by the University of California, San Francisco Institutional Review Board. The study followed the Strengthening the Reporting of Observational Studies in Epidemiology (STROBE) reporting guideline.

**Figure 1.**
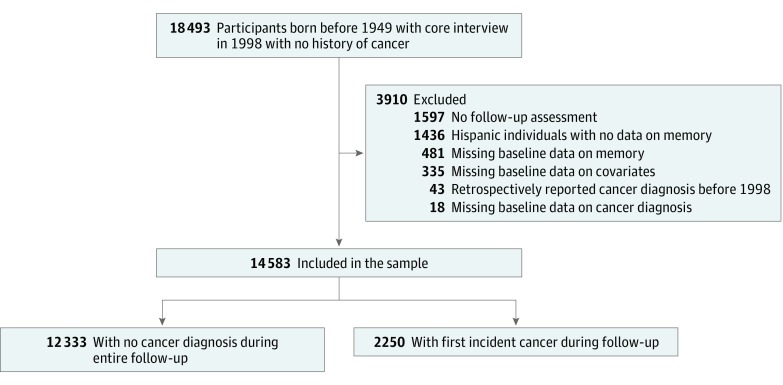
Selection of the Study Sample From the Health and Retirement Study Participants with Hispanic ethnicity were excluded because prior research indicated that the memory score calculation was not reliable for this group.

Information on participants was collected by telephone or in-person biennial interviews or proxy interviews for participants who were too impaired to answer. The analysis used the 1998 survey as baseline and included follow-up data through 2014, restricted to participants with no history of cancer at baseline who were born in 1948 or earlier. Of 18 493 eligible participants, we excluded 1436 participants with Hispanic ethnicity because prior research indicated the memory score calculation was not reliable for this group.^20^ We additionally excluded 481 participants with missing baseline data on memory, 18 with cancer, 335 with any of the covariates, and 1597 with no follow-up interviews. We also excluded 43 participants who retrospectively reported a cancer diagnosis before 1998. The final analytical sample included 14 583 participants (Figure 1). Biennial assessments were performed for up to 16 years from 1998 to 2014. Data analysis was performed from January 8 to October 5, 2018.

### Memory Function and Decline Outcomes

Memory in the HRS was assessed by immediate and delayed recall of a 10-word list, and proxy assessments were used for individuals too impaired to participate directly in memory assessments.^21^ Proxies, typically spouses, were asked to assess the participants’ memory on a 5-item Likert scale and to complete the validated 16-item Informant Questionnaire for Cognitive Decline.^22^ In the HRS, approximately 9% of interviews were with a proxy respondent during each wave (18% for those aged ≥80 years).^23,24^ We used a previously developed composite memory score that combined proxy and direct memory assessments for analyses of longitudinal data^20^ based on the Aging, Demographics, and Memory Study subsample of HRS participants who had in-person clinical assessments with comprehensive neuropsychological examinations during 2001 to 2003. This composite memory score was standardized using its 1998 mean and SD so that each unit change in memory score corresponded to approximately 1 SD in the baseline sample.

### Cancer Ascertainment

The HRS assessed cancer diagnosis at each interview wave with the question, “Has a doctor ever told you that you have cancer or a malignant tumor, excluding minor skin cancers?” Incident cancer was defined as a newly reported physician diagnosis of cancer. Participants who reported cancer were asked about month and year of diagnosis. We used the RAND Corporation HRS files,^25^ which provide a clean version of the self-reported new cancer diagnosis variable coded to incorporate previous and current interview responses of the HRS.^26^ We categorized participants into 2 groups: (1) participants with incident cancer (n = 2250), who reported a first diagnosis during the follow-up period, and (2) participants who remained cancer free (n = 12 333), for whom no cancer diagnosis was reported during the entire follow-up.

For incident cancer cases, we calculated time with respect to the initial cancer diagnosis, defining the date of diagnosis as time zero. The prediagnosis memory assessments were assigned negative time (in years) until the diagnosis and describe the precancer memory trajectory experienced in the years leading up to diagnosis. The postdiagnosis memory assessments were assigned positive time (in years) since diagnosis and describe the postcancer memory trajectory in the years after diagnosis. Separate slopes were estimated for the precancer and postcancer trajectories. We also included a time-dependent variable for whether the individual was diagnosed with cancer at the present or any prior wave (*cancer now*) to describe memory changes at the time of or shortly after diagnosis, as might be expected with treatment. People who did not report cancer were assigned values of zero for the precancer and postcancer time slopes and the *cancer now* variable. To retain participants with incomplete dates of diagnosis (n = 238), we used the midpoint between the last cancer-free interview date and the date when cancer was first reported.

### Other Covariates

We specified 2 sets of covariates based on the level of certainty of their potential confounding effect. The first set of confounders included variables (sex, race, and southern US birthplace [Delaware, Maryland, Washington, DC, Virginia, West Virginia, North Carolina, South Carolina, Georgia, Florida, Kentucky, Tennessee, Alabama, Mississippi, Arkansas, Louisiana, Oklahoma, or Texas]) that have been associated with cancer or memory function and decline but with no possibility of being influenced by an incipient cancer or prior memory level. The second set of covariates included childhood socioeconomic status using a previously validated index^27^; years of education; baseline household wealth; childhood self-rated health, using a 5-item Likert scale that ranged from poor to excellent health (categorized as poor or fair, good, or very good or excellent); baseline measures of vigorous physical activity, tobacco use, and alcohol use (none, low risk, and binge definitions from the National Institute on Alcohol Abuse and Alcoholism^28^); body mass index (calculated as weight in kilograms divided by height in meters squared); and self-reported history of comorbidities (hypertension, diabetes, heart disease, stroke, lung disease, and arthritis). Unless otherwise noted, continuous variables were centered at the sample mean.

### Statistical Analysis

Differences in baseline characteristics between participants with a new cancer diagnosis and those with no cancer diagnosis during follow-up were described with Pearson χ^2^ statistics for categorical variables, *t* tests for normally distributed continuous variables, and Wilcoxon rank sum tests for continuous variables without a normal distribution. Data were hierarchical, with each participant having up to 9 measurements of memory function at different ages. Thus, we used linear mixed-effects models to describe longitudinal memory score trajectories. Following the analytic model of Wang et al,^29^ age at each interview was used as the primary timescale, centered at 75 years of age (slightly older than the mean age across all follow-ups). Individual participants’ slopes of memory change with age and intercepts of memory slope at 75 years of age were allowed to vary as random effects. The covariance structure of random effects was coded as unstructured, allowing the correlation between intercepts and slopes to be estimated, and restricted maximum likelihood estimation was used. Wald χ^2^ tests were used to test the hypothesis of a differential rate of memory decline between the groups before and after the diagnosis. Stata/SE, version 15.1 (StataCorp) was used for the analysis.

We estimated memory change over time for participants who remained cancer free using updated age at each interview. We aimed to estimate differences in memory function between participants who developed cancer and participants who did not; we compared memory trajectories before diagnosis, short-term changes in memory function around the time of diagnosis, and long-term trajectories in memory after diagnosis. The time-dependent variable term *cancer now* accounted for the possible change in memory function around the time of diagnosis explained by events associated with the diagnosis (eg, initiation of cancer treatment, symptom management, or effects of stress). We included an interaction term between continuous time (with respect to cancer diagnosis) and the time-dependent *cancer now* variable, allowing for separate slopes in memory leading up to the cancer diagnosis and after cancer diagnosis. Models included age at cancer diagnosis centered at 75 years (set to 0 for all observations for participants who remained cancer free), which allowed comparison of memory function at the time of diagnosis with that in cancer-free individuals of the same age. Because age at each interview and age of cancer diagnosis were centered at 75 years, the intercept for this model compared the hypothesized memory score in a 75-year-old individual who never had cancer with memory in a 75-year-old individual immediately before he or she was diagnosed with cancer. A quadratic form of age was included to allow for curvilinear trajectories of memory based on the Methods in Longitudinal Dementia Research initiative guidelines for dementia research.^30^ Our first model (model 0) included as variables only the cancer variables, age, and interactions. The next model was adjusted for sex, race, and southern birthplace. The final, fully adjusted model included all other baseline covariates hypothesized as confounders. We conducted sensitivity analyses to assess the potential for survival bias by restricting the analysis to individuals who remained in the sample for at least 3, 4, or 5 waves. Cancer diagnosis could have been reported during any of the waves.

**Figure 2.**
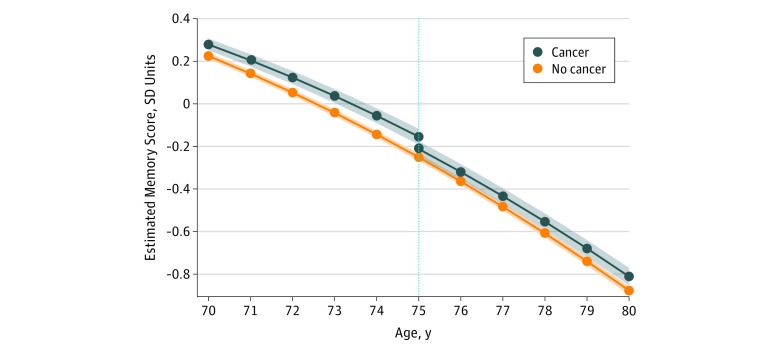
Memory Score Trajectories From Linear Mixed Models Estimated trajectories and 95% CIs (shaded area) of a hypothetical person with cancer diagnosis at 75 years of age (vertical line) in the reference categories of covariates (white race, female, 12 years of education, no history of alcohol use, no tobacco use, and no baseline comorbidities) compared with memory trajectories of a person with the same characteristics who remained cancer free. Memory score was standardized to its baseline mean (SD). Each unit in the memory score corresponds to approximately 1 SD in the baseline sample: *y*_ij_ = β_0_ + β_1_*x*_1_ + β_2_*x*_2_ + β_3_*x*_3_ + β_4_*x*_4_ + β_5_*x*_5_ + β_6_*x*_6_ + β_7_*x*_7_ + ∑β_k_*x*_k_, where *y* is the memory score for an individual *i* at *j* time, *x*_1_ is an indicator for having cancer diagnosis at any point in follow-up, *x*_2_ is an indicator for whether cancer has been diagnosed at *j* time (cancer now variable), *x*_3_ is *j* time minus time of diagnosis (number of years, always 0 for individuals who remained cancer free), *x*_4_ is years after diagnosis (number of years for people previously diagnosed with cancer, 0 if cancer had not yet been diagnosed, always 0 for individuals who remained cancer free), *x*_5_ is age at cancer diagnosis centered at 75 years (0 for individuals who remained cancer free), *x_6_* is age at each interview centered at 75 years, and *x_7_* is quadratic age at each interview centered at age 75 years, and *x_k_* is each of *k* baseline covariates. The estimated regression coefficients (β) are given in Table 2 and eTable 2 in the Supplement.

For clarity, we plotted model-estimated values for a hypothetical white woman with 12 years of education, no history of tobacco use, no alcohol use, and no comorbidities at baseline who was diagnosed with cancer at 75 years of age compared with the hypothesized memory trajectory for a person with the same characteristics but with no cancer diagnosis (Figure 2). The eFigure in the Supplement shows the observed memory scores with different ages by cancer status.

## Results

A total of 14 583 participants were included in the sample (mean [SD] age, 66.4 [10.4] years; 8453 [58.0%] female). Participants’ baseline characteristics are given in Table 1. A comparison of HRS participants with or without complete baseline data is given in eTable 1 in the Supplement. The mean (SD) follow-up was 11.5 (5.1) years (median, 14.1 years; interquartile range, 6.5-15.9 years); 2250 had a cancer diagnosis during follow-up, and 12 333 had no cancer diagnosis during follow-up. The mean (SD) age at cancer diagnosis was 71.7 (8.6) years. The crude incidence rate of cancer was 1439 per 100 000 person-years.

**Table 1.** Baseline Characteristics of Participants Who Were Cancer Free in 1998, Health and Retirement Study, United States^a^

Characteristic	Total (N = 14 583)	Participants With No Incident Cancer During Follow-up (n = 12 333)	Participants With First Incident Cancer During Follow-up (n = 2250)	2-Sided *P* Value
Age, mean (SD), y	66.4 (10.4)	66.0 (10.2)	64.8 (8.5)	<.001
Female	8453 (58.0)	7361 (59.7)	1092 (48.5)	<.001
Nonwhite race	2480 (17.0)	2159 (17.5)	321 (14.3)	<.001
Length of education, median (IQR), y	12 (11-14)	12 (11-14)	12 (12-15)	<.001
Childhood SES index, mean (SD)	0.07 (0.9)	0.07 (0.9)	0.12 (0.9)	.01
Southern birthplace	5365 (36.8)	4593 (37.2)	772 (34.3)	.008
Household wealth per $10 000, median (IQR)	14.1 (4.8-34.5)	13.8 (4.5-33.9)	16.8 (5.8-38.3)	<.001
BMI, mean (SD)	26.9 (5.1)	26.9 (5.2)	27.3 (5.0)	<.001
Vigorous physical activity	6601 (45.3)	5492 (44.5)	1109 (49.3)	<.001
Current smoking	2418 (16.6)	2012 (16.3)	406 (18.0)	.04
Alcohol use				
Low risk	4198 (28.7)	3446 (27.9)	752 (33.4)	<.001
Binge	305 (2.1)	241 (2.0)	64 (2.8)	
Childhood self-rated health				
Low	888 (6.1)	760 (6.2)	128 (5.7)	.11
Good	2666 (18.3)	2285 (18.5)	381 (16.9)	
High	11 029 (75.6)	10 463 (75.1)	1741 (77.4)	
Hypertension	6130 (42.0)	5188 (42.1)	942 (41.9)	.86
Diabetes	1706 (11.7)	1479 (12.0)	227 (10.1)	.01
Heart disease	2788 (19.1)	2386 (19.4)	402 (17.9)	.10
Stroke	896 (6.1)	773 (6.3)	123 (5.5)	.15
Lung disease	922 (6.3)	771 (6.3)	151 (6.7)	.41
Arthritis	7018 (48.1)	5957 (48.3)	1061 (47.2)	.32

Abbreviations: BMI, body mass index (calculated as weight in kilograms divided by height in meters squared); IQR, interquartile range; SES, socioeconomic status.

^a^ Data are presented as number (percentage) of participants unless otherwise indicated.

Before diagnosis, participants who were subsequently diagnosed with cancer had higher memory scores than those who were not diagnosed (Figure 2). For example, the mean memory function at 75 years of age for individuals who were not diagnosed with cancer during follow-up was −0.251 SD units (95% CI, −0.269 to −0.233 SD units), whereas the mean memory function for participants who reported a new cancer diagnosis at 75 years of age was −0.155 SD units (95% CI, −0.191 to −0.118 SD units) immediately before diagnosis. Therefore, memory function was 0.096 SD units (95% CI, 0.060-0.133 SD units) higher in individuals just before a cancer diagnosis at 75 years of age compared with that in individuals of the same age without a cancer diagnosis. In addition, cancer diagnosis was associated with a decline in memory in the short term after the diagnosis (−0.058 SD units [95% CI, −0.084 to −0.032 SD units]).

Before diagnosis, individuals who were subsequently diagnosed with cancer experienced slower memory decline compared with those of similar ages who remained free of diagnosis. For example, between the ages of 65 and 75 years, participants who were not diagnosed with cancer had a mean decline in memory of 0.801 SD units (95% CI, −0.810 to −0.792 SD units); this decline was 0.084 SD units (95% CI, 0.049-0.119 SD units) less in participants who were subsequently diagnosed with cancer. That is, before diagnosis, individuals diagnosed with cancer were experiencing memory decline at a 10.5% (95% CI, 6.2%-14.9%) slower rate than similar individuals who remained free of a cancer diagnosis. Between 75 and 85 years of age, participants who remained free of cancer diagnosis experienced a mean decline in memory of 1.404 SD units (95% CI, −1.418 to −1.389 SD units); the mean decline in this age group was 0.055 SD units (95% CI, 0.012-0.097 SD units) less in participants previously diagnosed with cancer. This difference represents a 3.9% (95% CI, 0.9%-6.9%) slower rate of memory decline after a cancer diagnosis compared with similar individuals who remained free of a cancer diagnosis (Figure 2). These trends in memory scores were calculated using the coefficients of the fully adjusted model presented in Table 2. The covariate coefficients are presented in eTable 2 and eTable 3 in the Supplement.

**Table 2.** Estimated Regression Coefficients From Linear Mixed Models for Memory Change (SD Units) per Decade, Health and Retirement Study, United States, 1998-2014

Characteristic	β (95% CI)^a^			
	Model 0	Model 1^b^	Model 2^c^
Participants who remained cancer free			
Memory slope with linear age	−1.115 (−1.127 to −1.104)	−1.116 (−1.126 to −1.105)	−1.102 (−1.113 to −1.091)
Memory slope with quadratic age	−0.309 (−0.314 to −0.303)	−0.295 (−0.300 to −0.290)	−0.301 (−0.306 to −0.296)
Difference for people diagnosed with cancer			
Precancer memory slope (linear)	0.075 (0.037 to 0.113)	0.091 (0.056 to 0.126)	0.084 (0.049 to 0.119)
Postcancer memory slope (linear)	0.058 (0.013 to 0.102)	0.071 (0.028 to 0.113)	0.055 (0.012 to 0.097)
Change in memory at the time of diagnosis	−0.056 (−0.081 to −0.297)	−0.057 (−0.082 to −0.030)	−0.058 (−0.084 to −0.032)

^a^ β values are regression coefficients for memory change per decade before and after diagnosis and at the time of diagnosis among participants with cancer compared with memory change per decade among those who remained cancer free throughout follow-up. Two-sided *P* < .01 for all comparisons.

^b^ Adjusted for sex, race, and Southern birthplace.

^c^ Adjusted for sex, race, Southern birthplace, childhood socioeconomic status, length of education, baseline household wealth, childhood self-rated health, alcohol use, current smoking, body mass index, baseline history of hypertension, diabetes, heart disease, stroke, lung disease, and arthritis.

Sensitivity analyses restricting the sample to individuals who had additional waves of follow-up found similar associations between prediagnosis memory trajectories and short-term decline in memory after cancer diagnosis, albeit with wider CIs in the smaller sample. The difference in rate of postdiagnosis memory decline and age-associated memory decline was not statistically significant in the smaller sample (eTable 4 in the Supplement).

## Discussion

In this large cohort, middle-aged and older US adults who reported an incident cancer diagnosis experienced slower memory decline than individuals who were not diagnosed with cancer. Mean memory function was better among individuals with a cancer diagnosis at all time points during follow-up, even in the immediate short-term period after diagnosis, compared with otherwise similar individuals who remained free of a cancer diagnosis. The group with incident cancer experienced a short-term decline in memory in the period immediately after diagnosis, and the difference in memory function between the 2 groups narrowed. After diagnosis, the mean decline in memory continued at a rate similar to that before diagnosis; thus, the difference in memory function between the groups increased slightly with each additional year after cancer diagnosis.

This study supports the previously reported inverse association between cancer and AD. First, the slower memory decline before cancer diagnosis in the group with incident cancer suggests that factors preceding or coinciding with carcinogenesis might also be associated with a decreased risk of AD. Common genetic risk factors for cancer and AD may explain the inverse comorbidity.^31^ The cellular environment is key in the pathogenesis of both conditions; local or systemic signals that promote tumor cell growth in peripheral cells could translate into antiapoptotic signals in neurons.^32^ Elucidation of these potential common factors associated with cancer and dementia could contribute to developing preventive and treatment strategies for AD. However, replication of these findings in other cohorts with multiple assessment of cognitive function over time is warranted because, to our knowledge, this is the first study to evaluate long-term cognitive trajectories before and after a cancer diagnosis. Second, we captured the short-term association of a cancer diagnosis with memory functioning, which has previously been reported in neuropsychological studies^14,15,16^ among patients with breast and prostate cancers. The present study was one of the first to date to use a population-based rather than clinic-based sample to contribute to the literature on cancer-related cognitive decline,^33,34^ which reported that memory function declined after diagnosis regardless of cancer type or treatment received in a nationwide sample of adults 50 years or older. This proximal association of cancer and short-term cognitive decline was initially hypothesized to be associated with the cytotoxic action of chemotherapy and other treatment modalities, such as radiotherapy or immunotherapy. Because we found that cancer diagnosis was associated with a deterioration in memory function at the population level, other potential mechanisms may be associated with this short-term memory decline, including chronic pain and symptom management during cancer treatment, comorbidities, pathologic stress, or changes in socioeconomic status related to medical expenses.^16,33,35,36^

Our study addressed the potential for survival bias and detection bias using assessments of memory function at different ages (or years of follow-up) in all individuals included in the sample regardless of their cancer status. If more vulnerable individuals with a higher risk of AD were experiencing more deaths, our study design should have detected an accelerated memory decline in the years before their death. This study did not estimate the incidence of AD in the group with cancer or the comparison group because this estimation would introduce selection bias caused by competing risks. A slower long-term decline in memory has been reported to be associated with a slower progression along the continuum of mild cognitive impairment and AD.^37^ Thus, our results are consistent with previous literature on the association between cancer and AD. Although the memory advantage associated with incident cancer was modest on a continuous scale, the 0.049–SD unit difference in mean memory function between the group with incident cancer and the comparison group at 2 years after diagnosis would translate into an approximately 0.90 risk ratio for memory impairment in the group with cancer compared with a population with a 5% prevalence of impairment (eAppendix 2 in the Supplement).

### Limitations

This study has several limitations. Potential survival bias attributable to differential follow-up times is a concern in our interpretation of the results. In the sensitivity analysis, we obtained similar results when we compared individuals with similar length of follow-up by restricting our sample to those with at least 3, 4, or 5 waves, although this merits confirmation in independent data sources. Misclassification of cancer diagnosis status was also possible in our study because we used self-reported data on cancer diagnosis with no medical record verification because even major diagnoses are not perfectly reported.^38,39,40^ Although proxy reports of cancer diagnosis were included, our study cannot rule out the possibility that people with worse memory differentially underreported cancer diagnoses. However, previous studies^4,41^ of this topic used administratively recorded cancer diagnoses, suggesting that this is not a plausible explanation for the inverse association between cancer and AD. Participants classified as cancer free may have had undiagnosed cancer, but this scenario would tend to decrease the differences in memory function between the groups.

Our estimate of memory decline immediately after cancer diagnosis was imprecise because the assessments of memory occurred every 2 years; thus, the immediate lag between diagnosis and memory assessment varied up to a maximum of less than 2 years after diagnosis. Furthermore, we did not have information available on cancer type, staging, and treatment potentially relevant to memory decline. Verbal memory function was the only cognitive domain routinely assessed in all HRS participants 50 years or older. Although memory impairment is the hallmark of AD and dementia, patients with this condition also manifest decline in other cognitive domains.^42^

## Conclusions

In this population-based cohort study of middle-aged and older US adults, individuals with an incident cancer diagnosis had better memory function and slower memory decline before and after their diagnosis compared with similarly aged individuals who remained cancer free during the follow-up period. These novel findings support the possibility of a common pathologic process working in opposite directions in cancer and AD. Identification of a potential association between carcinogenesis and neurodegeneration may open a new avenue in research for prevention and treatment of AD.

## Notes

Supplement. **eAppendix 1.** List of Health and Retirement Study Datasets Used for This Study **eTable 1.** Comparison of Health and Retirement Study Participants With and Without Complete Baseline Data **eFigure.** LOWESS Curves of the Observed Memory Scores in Participants From the Health and Retirement Study **eTable 2.** Coefficients of the Fully Adjusted Model of Memory **eTable 3.** Coefficients of the Fully Adjusted Model of Memory With Interactions of Baseline Covariates and Age **eTable 4.** Estimated Regression Coefficients of Memory Function and Memory Decline Restricting the Sample to Those Who Had at Least 3, 4, or 5 Follow-up Waves **eAppendix 2.** Estimation of Risk Ratio for Memory Impairment Between Participants With Cancer Diagnosis (2 Years After Cancer) and Similar Aged Participants With No Cancer Diagnosis During Follow-up
