# Supplementary material for: Rate of Memory Change Before and After Cancer Diagnosis
Source: JAMA Netw Open. 2019 Jun 21;2(6):e196160. doi: 10.1001/jamanetworkopen.2019.6160 (PMC6593639; doi:10.1001/jamanetworkopen.2019.6160)
Supplement: Supplement. — eAppendix 1. List of Health and Retirement Study Datasets Used for This Study eTable 1. Comparison of Health and Retirement Study Participants With and Without Complete Baseline Data eFigure. LOWESS Curves of the Observed Memory Scores in Participants From the Health and Retirement Study eTable 2. Coefficients of the Fully Adjusted Model of Memory eTable 3. Coefficients of the Fully Adjusted Model of Memory With Interactions of Baseline Covariates and Age eTable 4. Estimated Regression Coefficients of Memory Function and Memory Decline Restricting the Sample to Those Who Had at Least 3, 4, or 5 Follow-up Waves eAppendix 2. Estimation of Risk Ratio for Memory Impairment Between Participants With Cancer Diagnosis (2 Years After Cancer) and Similar Aged Participants With No Cancer Diagnosis During Follow-up [file jamanetwopen-2-e196160-s001.pdf]

Supplementary Online Content

Ospina-Romero M, Abdiwahab E, Kobayashi L, et al. Rate of memory change before and after cancer diagnosis. *JAMA Netw Open*. 2019;2(6):e196160. doi:10.1001/jamanetworkopen.2019.6160

eAppendix 1. List of Health and Retirement Study Datasets Used for This Study

eTable 1. Comparison of Health and Retirement Study Participants With and Without Complete Baseline Data

eFigure. LOWESS Curves of the Observed Memory Scores in Participants From the Health and Retirement Study

eTable 2. Coefficients of the Fully Adjusted Model of Memory

eTable 3. Coefficients of the Fully Adjusted Model of Memory With Interactions of Baseline Covariates and Age

eTable 4. Estimated Regression Coefficients of Memory Function and Memory Decline Restricting the Sample to Those Who Had at Least 3, 4, or 5 Follow-up Waves

eAppendix 2. Estimation of Risk Ratio for Memory Impairment Between Participants With Cancer Diagnosis (2 Years After Cancer) and Similar Aged Participants With No Cancer Diagnosis During Follow-up

This supplementary material has been provided by the authors to give readers additional information about their work.

© 2019 Ospina-Romero M et al. *JAMA Network Open.*

eAppendix 1. List of Health and Retirement Study Datasets Used for This Study

1. RAND HRS Data Documentation. Version P

2. Tracker file HRS. Version 2014

3. HRS core dataset from 2000 to 2014 (for year and month of cancer diagnosis)

4. Imputations of memory score dataset (Wu et al. 2013)

5. Childhood socioeconomic measures dataset (Vable et al. 2017)

6. HRS core dataset 1998 (for childhood self-rated health)

© 2019 Ospina-Romero M et al. *JAMA Network Open.*

eTable 1. Comparison of Health and Retirement Study participants with and without complete baseline data. Participants with missing baseline data were mainly from Hispanic origin with low educational level.

	Complete		Missing	
Characteristic	n	(%)	n	(%)
Total	16,223	(87.7)	2,270	(12.3)
Age, mean (SD), years	66.4	(10.4)	64.7	(10.4)
Male	6,924	(42.7)	1,006	(44.3)
Race				
White	13,409	(82.6)	1,729	(76.2)
Black	2,470	(15.2)	224	(9.9)
Other	344	(2.1)	311	(13.7)
Hispanic	32	(0.2)	1,437	(63.4)
Education				
Less than high school	4,159	(25.6)	1,238	(54.5)
High school	8,643	(53.3)	778	(34.3)
Some/complete college	2,186	(13.5)	176	(7.8)
Master or higher degree	1,235	(7.6)	78	(3.4)

© 2019 Ospina-Romero M et al. *JAMA Network Open.*

eFigure. LOWESS Curves of the Observed Memory Scores in Participants From the Health and Retirement Study. A. The trajectories of memory function in the cancer group before the diagnosis and the comparison group are diverging at older ages. B. The trajectories of memory function in the cancer group after the diagnosis and the comparison group are diverging at older ages. Bandwidth = 0.2.

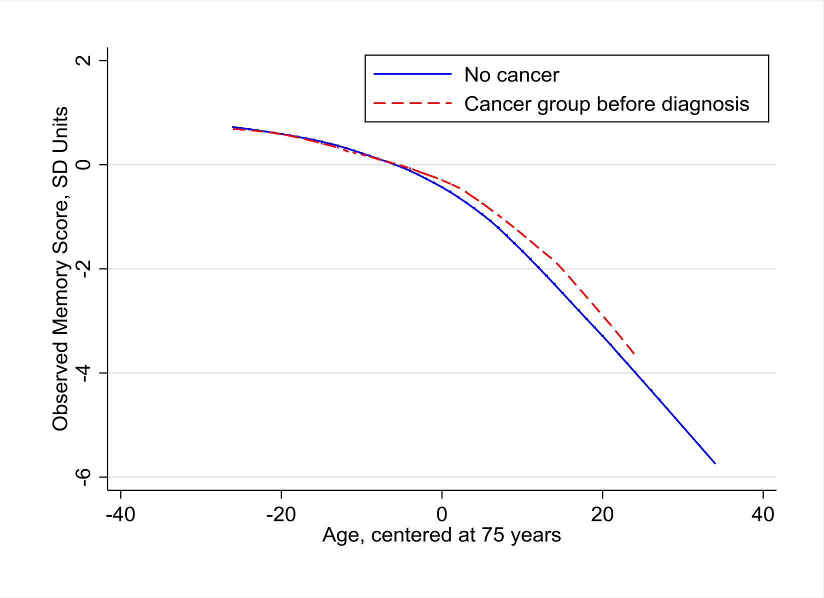

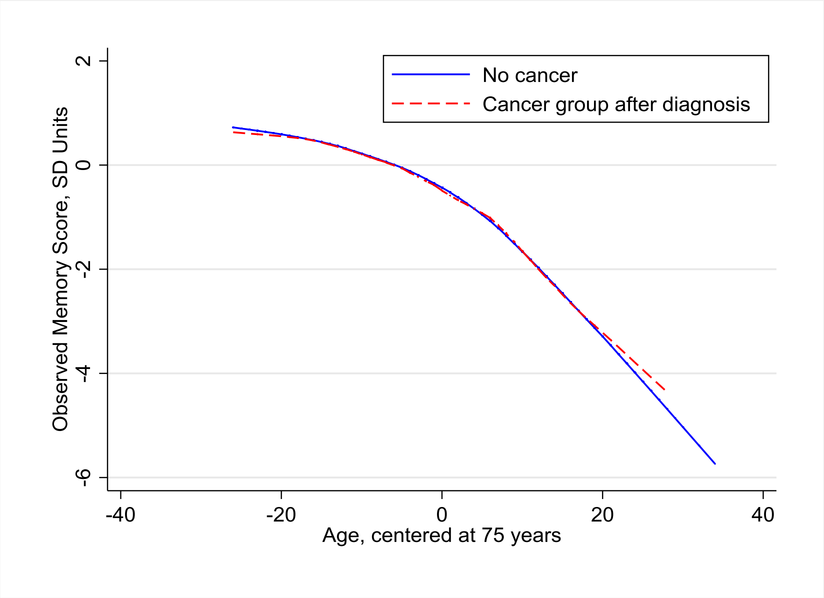

© 2019 Ospina-Romero M et al. *JAMA Network Open.*

**eTable 2. Coefficients of the Fully Adjusted Model of Memory**				
Characteristic		β	95% CI	P
Average memory score at age 75 (constant term)		-0.251	(-0.269, -0.233)	< 0.001
Difference in memory score right before cancer		0.096	(0.060, 0.1312)	< 0.001
Change in memory at the time of diagnosis		-0.058	(-0.084, -0.032)	< 0.001
Participants who remained cancer-free				
Memory slope with linear age (years)		-0.110	(-0.111, -0.110)	< 0.001
Memory slope with quadratic age ($years^2$)		-0.003	(-0.0031, -0.0030)	< 0.001
Difference in rate of change for people diagnosed with cancer compared to those who remained cancer-free				
Pre-cancer memory slope (linear, years)		0.008	(0.005, 0.012)	< 0.001
Post-cancer memory slope (linear, years)		0.005	(0.001, 0.010)	0.012
Covariates				
Male gender		-0.343	(-0.355, -0.332)	< 0.001
Nonwhite race		-0.651	(-0.666, -0.635)	< 0.001
Years of education (center at 12 years)		0.032	(0.030, 0.034)	< 0.001
Southern birth place		-0.067	(-0.079, -0.050)	< 0.001
Childhood self-rated health (reference: very good/excellent)	Good	-0.025	(-0.040, -0.010)	0.001
	Poor/Fair	-0.027	(-0.051, -0.003)	0.024
Childhood SES score		-0.008	(-0.014, -0.002)	0.013
Total household wealth (per 100,000)		-0.332	(-0.792, 0.127)	0.156
Current tobacco use		-0.008	(-0.022, 0.006)	0.267
Current alcohol use (reference: none)	Low risk	0.007	(-0.006, 0.019)	0.301
	Binge	-0.027	(-0.061, 0.008)	0.131
Vigorous physical activity		0.023	(0.012, 0.034)	< 0.001
Body Mass Index		0.002	(0.001, 0.003)	0.001
Comorbidities				
Hypertension		-0.020	(-0.032, -0.008)	0.001
Diabetes mellitus		-0.039	(-0.058, -0.020)	< 0.001
Lung Disease		-0.001	(-0.028, 0.025)	0.920
Heart Disease		-0.009	(-0.026, 0.009)	0.328
Stroke		-0.063	(-0.093, -0.032)	< 0.001
Arthritis		-0.010	(-0.021, 0.002)	0.097

© 2019 Ospina-Romero M et al. *JAMA Network Open.*

eTable 3. Coefficients of the Fully Adjusted Model of Memory With Interactions of Baseline Covariates and Age				
Characteristic		β	95% CI	P
Average memory score at age 75 (constant term)		-0.193	(-0.221, -0.165)	< 0.001
Difference in memory score right before cancer		0.084	(0.048, 0.120)	< 0.001
Change in memory at the time of diagnosis		-0.056	(-0.081, -0.030)	< 0.001
Participants who remained cancer-free				
Memory slope with linear age (years)		-0.105	(-0.107, -0.103)	< 0.001
Memory slope with quadratic age ($years^2$)		-0.003	(-0.0030, -0.0029)	< 0.001
Difference for people diagnosed with cancer				
Pre-cancer memory slope (linear, years)		0.008	(0.004, 0.011)	< 0.001
Post-cancer memory slope (linear, years)		0.004	(-0.031, 0.080)	0.069
Covariates				
Male gender		-0.398	(-0.421, -0.374)	< 0.001
Nonwhite race		-0.768	(-0.801, -0.735)	< 0.001
Years of education (center at 12 years)		0.056	(0.051, 0.060)	< 0.001
Southern birth place		-0.136	(-0.161, -0.110)	< 0.001
Childhood self-rated health (reference: high)	Good	-0.047	(-0.076, -0.018)	0.002
	Low	-0.043	(-0.091, 0.004)	0.072
Childhood SES score		-0.012	(-0.026, 0.003)	0.115
Total household wealth (per 100,000)		2.920	(1.42, 4.43)	< 0.001
Current tobacco use		-0.119	(-0.151, -0.086)	< 0.001
Current alcohol use (reference: none)	Low risk	0.005	(0.003, 0.007)	< 0.001
	Binge	-0.011	(-0.017, -0.005)	< 0.001
Vigorous physical activity		0.071	(0.048, 0.094)	< 0.001
Body Mass Index		0.000	(-0.002, 0.003)	0.710
Comorbidities				
Hypertension		-0.020	(-0.043, 0.004)	0.103
Diabetes mellitus		-0.151	(-0.188, -0.115)	< 0.001
Lung Disease		-0.010	(-0.058, 0.037)	0.669
Heart Disease		-0.005	(-0.034, 0.024)	0.754
Stroke		-0.203	(-0.250, -0.156)	< 0.001
Arthritis		0.012	(-0.011, 0.035)	0.300
Interactions				
Education*age		0.0023	(0.002,0.003)	<0.001
Nonwhite*age		-0.0099	(-0.012, -0.007	<0.001
Male*age		-0.0053	(-0.007, -0.004)	<0.001
SUS*age		-0.0057	(-0.007, -0.004)	<0.001
Childhood SES*age		-0.0005	(-0.001, 0.000)	0.295
Total household wealth*age		0.2390	(0.134, 0.344)	<0.001
Childhood health*age	good	-0.0018	(-0.004, 0.000)	0.09
	low	-0.0013	(-0.005, 0.002)	0.442

© 2019 Ospina-Romero M et al. *JAMA Network Open.*

Smoking*age		-0.0079	(-0.010, -0.006)	<0.001
Alcohol*age	low risk	0.0053	(0.003, 0.007)	<0.001
	Binge	-0.0112	(-0.017, -0.005)	<0.001
Physical activity*age		0.0042	(0.003, 0.006)	<0.001
BMI*age		-0.0001	(-0.0002, 0.0001)	0.503
HTN*age		0.0004	(-0.001, 0.002)	0.614
Diabetes*age		-0.0096	(-0.012, -0.007)	<0.001
Lung Disease*age		-0.0011	(-0.005, 0.002)	0.54
Heart Disease*age		0.0003	(-0.002, 0.002)	0.758
Stroke*age		-0.0142	(-0.018, -0.011)	<0.001
Arthritis*age		0.0019	(0.00028, 0.0035)	0.021

© 2019 Ospina-Romero M et al. *JAMA Network Open.*

eTable 4. Estimated Regression Coefficients of Memory Function and Memory Decline Restricting the Sample to Those Who Had at Least 3, 4, or 5 Follow-up Waves

	Model 3 [a]		Model 4 [b]		Model 5 [c]	
Characteristic	β	95% CI	β	95% CI	β	95% CI
Memory function						
Average memory score at age 75 (constant term)	-0.221	(-0.239, -0.203)	-0.199	(-0.217, -0.181)	-0.169	(-0.187, -0.150
Difference in memory score right before cancer	0.075	(0.039, 0.112)	0.062	(0.026, 0.0.98)	0.048	(0.012, 0.084)
Change in memory at the time of diagnosis	-0.050	(-0.076, -0.024)	-0.038	(-0.065, -0.012)	-0.031	(-0.058, -0.045)
Memory decline per decade						
Among participants not diagnosed with cancer						
Memory slope with linear age	-1.088	(-1.099, -1.076)	-1.074	(-1.086, -1.063)	-1.058	(-1.070, -1.046)
Memory slope with quadratic age	-0.303	(-0.307, -0.297)	-0.302	(-0.307, -0.297)	-0.303	(-0.308, -0.298)
Difference for people diagnosed with cancer						
Pre-cancer memory slope (linear)	0.066	(0.031, 0.102)	0.055	(0.019, 0.090)	0.044	(0.009, 0.080)
Post-cancer memory slope (linear)	0.037	(-0.006, 0.079)	0.021	(-0.021, 0.063)	0.005	(-0.037, 0.046)

a. Included participants with 3 or more follow-up waves, n = 13,044

b. Included participants with 4 or more follow-up waves, n = 11,829

c. Included participants with 5 or more follow-up waves, n = 10,730

© 2019 Ospina-Romero M et al. *JAMA Network Open.*

eAppendix 2. Estimation of risk ratio for memory impairment between participants with cancer diagnosis (2 years after cancer) and similar aged participants with no cancer diagnosis during follow-up.

The mean memory function 2 years after cancer, when cancer diagnosis was at 75 years of age, is -0.43 units (95% CI: 0.47, 0.40). The mean memory function at 77 years of age in the group with no cancer diagnosis is -0.48 units (95% CI: 0.50, 0.46). The averaged difference in memory function 2 years after diagnosis was 0.049 units (95% CI: 0.012, 0.086).

If we assume that the risk of memory impairment in the population with no cancer diagnosis is 10%, we can find the cutoff level when memory becomes impaired (threshold for memory impairment). The z-score that cuts the lower 10% of the population with no cancer is -1.28. (see Figure below).

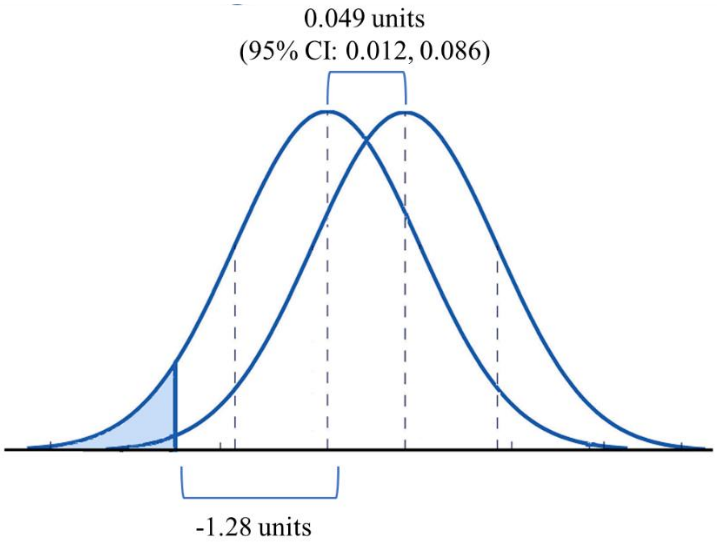

To estimate the risk of cognitive impairment in the cancer group if we applied the same threshold, we added the averaged difference of memory function between the two populations to the cut-off level in the population with no cancer diagnosis.

$$-1.28\,units - 0.049\,units = -1.33\,units$$

The risk of memory impairment in the population with 2 years history of cancer diagnosis is the area under the curve below the z-score of -1.33 which corresponds 9.2% of the population. In other words, the impairment threshold that classifies the lower 10% of the sample without cancer as "impaired" would classify only 9.2% of the sample who were diagnosed with cancer 2 years previously. This corresponds with a risk ratio for impairment of 0.92:

$$RR = \frac{Risk\ in\ the\ cancer\ group}{Risk\ group\ with\ no\ cancer} = \frac{0.092}{0.1} = 0.92$$

© 2019 Ospina-Romero M et al. *JAMA Network Open.*

Using a similar calculation, if the impairment threshold cuts off 5% of the sample without cancer, it would imply a RR of approximately 0.90 associated with a cancer diagnosis 2 years previously.

© 2019 Ospina-Romero M et al. *JAMA Network Open.*
